# Calciphylaxis in the Setting of Hemodialysis, Liver Cirrhosis, and Warfarin Therapy for Atrial Fibrillation: An Argument for Alternative Anti-Embolic Therapy

**DOI:** 10.7759/cureus.8014

**Published:** 2020-05-07

**Authors:** Vidya M Medepalli, Loretta S Davis, Lalitha C Medepalli, Sandeep A Padala

**Affiliations:** 1 Department of Medicine, Augusta University Medical College of Georgia, Augusta, USA; 2 Department of Dermatology, Augusta University Medical College of Georgia, Augusta, USA; 3 Cardiology, The Heart Center of Northeast Georgia Medical Center, Gainesville, USA; 4 Division of Nephrology, Hypertension, and Transplant, Augusta University Medical College of Georgia, Augusta, USA

**Keywords:** calciphylaxis, calcific uremic arteriopathy, cirrhosis, hemodialysis, warfarin therapy, paroxysmal atrial fibrillation, watchman, painful skin lesions, skin necrosis, diffuse soft tissue calcinosis

## Abstract

Calciphylaxis, also referred to as calcific uremic arteriolopathy, is a rare, life-threatening cutaneous systemic disease that typically occurs in the setting of end-stage renal disease (ESRD). ESRD is the most recognized risk factor for calciphylaxis but it is not the sole risk factor. Calciphylaxis has also been associated with liver cirrhosis with or without concurrent renal disease. The current case describes a patient who developed calciphylaxis in the setting of hemodialysis, liver cirrhosis, and atrial fibrillation managed with warfarin therapy, all risk factors for calciphylaxis. The need for alternatives to warfarin therapy, specifically in patients with atrial fibrillation on hemodialysis for ESRD who are at increased risk for calciphylaxis, is discussed. Specifically, the left atrial appendage occluder device is described and the need for interdisciplinary management of these patients is stressed.

## Introduction

Calciphylaxis, an uncommon cutaneous systemic condition due to occlusion of microvessels in the subcutaneous adipose tissue and dermis, carries a high morbidity and an estimated six-month mortality of 50% [1]. Patients with this condition present with painful livedo reticularis and violaceous plaques or indurated nodules which may result in cutaneous necrosis and ulceration. Calciphylaxis presenting with cardiac comorbidities, such as atrial fibrillation (AF), necessitating chronic anticoagulation represents an intersection of medical conditions without clear treatment guidelines. Providing therapeutic anticoagulation, such as warfarin, for these patients is extremely difficult due to the simultaneous presence of underlying end-stage renal disease (ESRD). The need for alternatives to warfarin therapy, specifically in patients with AF on hemodialysis for ESRD who are at increased risk for calciphylaxis, and the potential advantage of using the left atrial appendage occluder device are discussed.

## Case presentation

**Figure 1:**
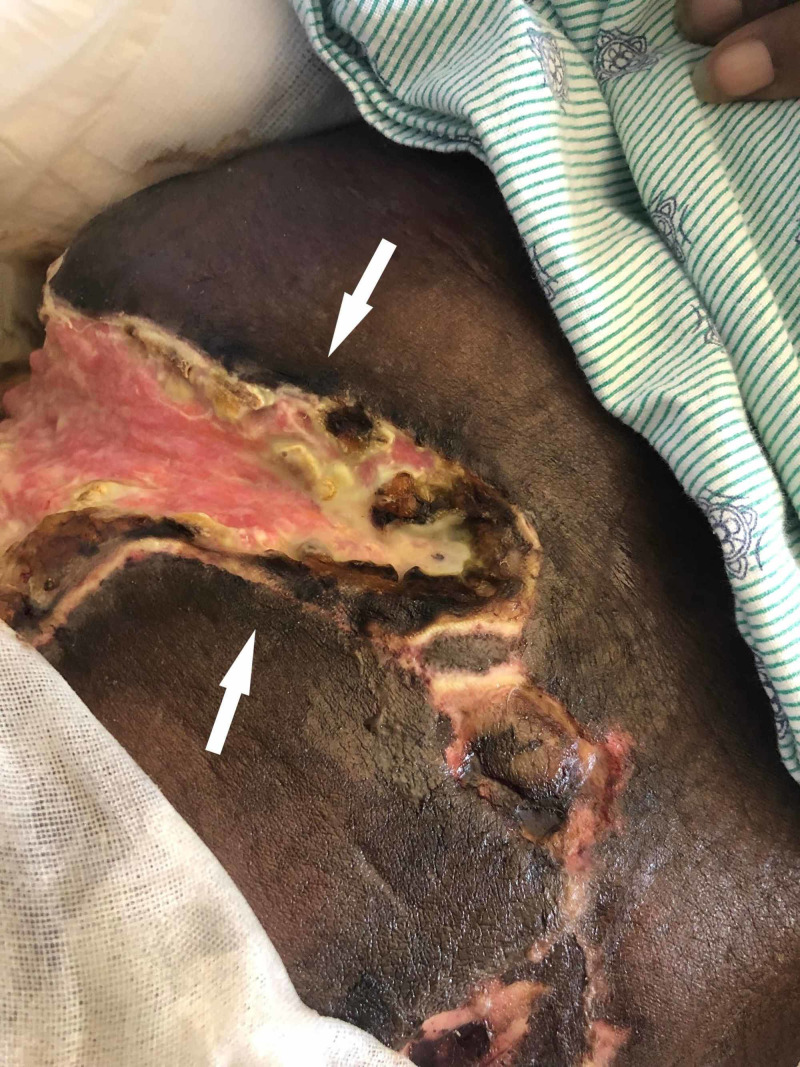
A broad tract of dusky hyperpigmentation, extremely firm induration, and overlying necrosis with ulceration (arrows) covered the left medial thigh

A 48-year-old African American woman was transferred to our facility for evaluation and management of abdominal pain. History included hypertension, ESRD on hemodialysis for the previous three years, paroxysmal AF on chronic warfarin therapy, and alcoholic cirrhosis with recurrent ascites. Over the preceding few days, she reported painful skin blistering and ulcerations diffusely over her body, worse on the left lower extremity. On physical examination, she was afebrile, thin, frail, and poorly nourished. She kept her knees bent and was unable to extend them due to pain. Broad, firm, necrotic plaques with ulcerations were present on the bilateral upper thighs and flanks, bilateral lower breasts, and lower abdomen (Figure 1). She had tenderness to palpation of the left hip and inner thighs. Her abdomen was distended, firm, exquisitely tender to palpation, and without hepatosplenomegaly. Bowel sounds were hypoactive. A mild fluid wave improved with paracentesis.

Laboratory findings included electrolyte abnormalities, elevated aspartate aminotransferase, alanine aminotransferase, alkaline phosphatase, and phosphorus, as well as a low albumin level (Table 1).

**Table 1:** Laboratory Data

Laboratory tests	Result (normal range)
White blood count	10.4 thousands/mm³ (4.5 to 11.0 thousands/mm³)
Hemoglobin	11.3 g/dL (11.5 - 15.5 g/dL)
Sodium	133 mEq/L (135 - 145 mEq/L)
Potassium	3.8 mEq/L (3.5 - 5.0 mEq/L)
Blood Urea Nitrogen	36 mg/dL (7 - 20 mg/dL)
Creatinine	3.6 mg/dl (0.7 - 1.3 mg/dl)
Calcium	9.3 mg/dl (9 - 10.5 mg/dl)
Albumin	2.8 g/dl (3.5 - 5.5 g/dl)
Aspartate Aminotransferase	164 U/L (0 - 35 units/L)
Alanine Aminotransferase	171 U/L (0 - 35 units/L)
Alkaline Phosphatase	392 units/L (36 - 92 units/L)
Total Bilirubin	0.8 mg/dL (0.2 - 1.2 mg/dL)
Phosphorus	9.2 mg/dL (3 - 4.5 mg/dl)
International Normalized Ratio (INR)	2.0 (0.8 - 1.1)
Lipase	48 mEq/L (1 - 160 mEq/L)

**Figure 2:**
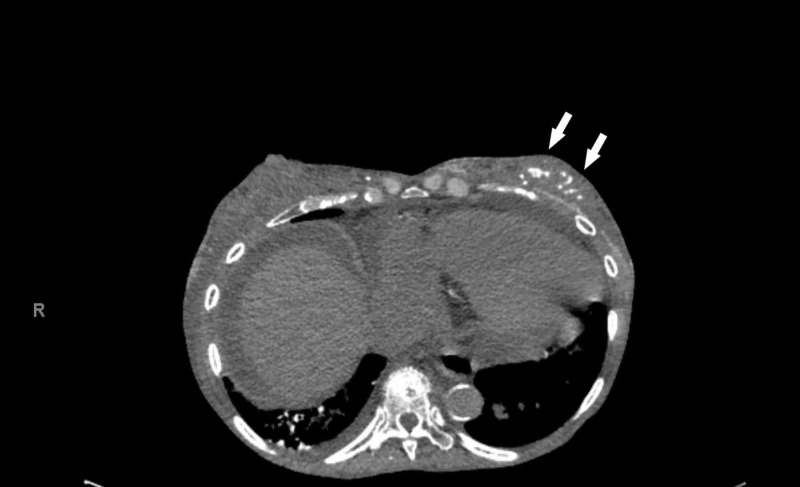
Computed tomography (CT) scan demonstrated soft tissue calcinosis (arrows)

Computed tomography (CT) of the abdomen and pelvis without contrast revealed diffuse soft tissue anasarca and increased density within the adipose tissue, representing diffuse soft tissue calcinosis (Figure 2).

**Figure 3:**
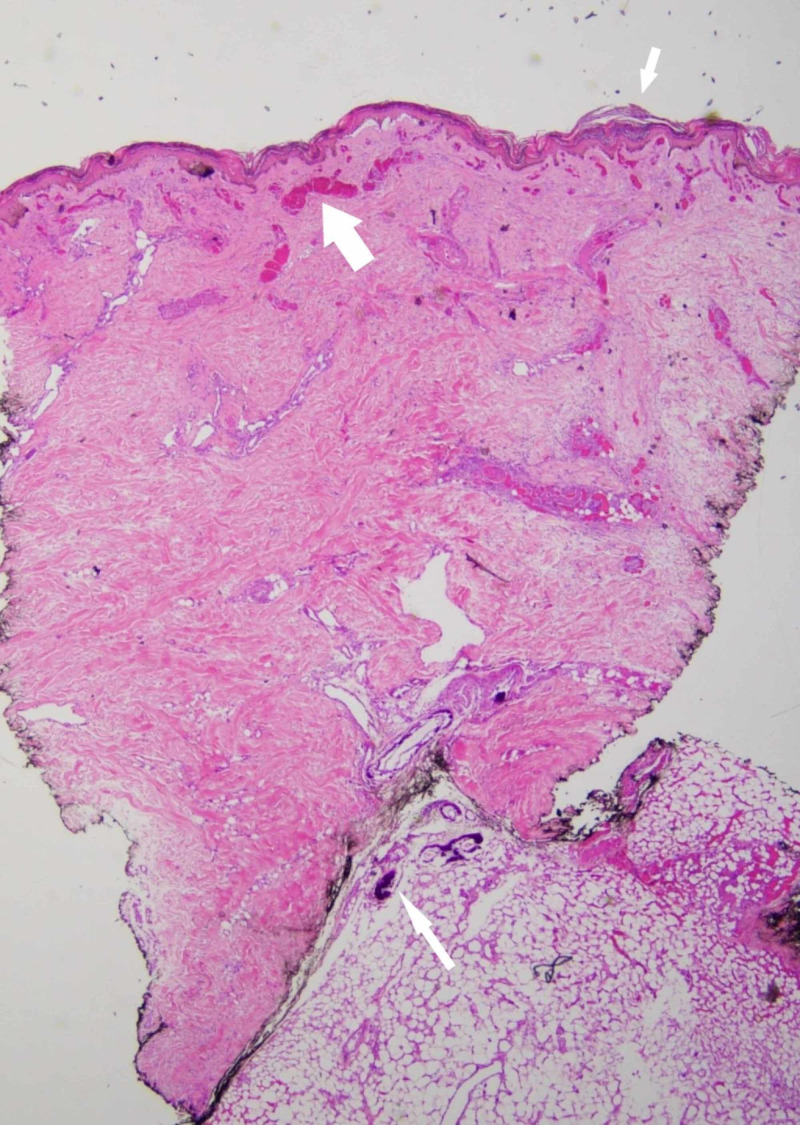
Low-power view demonstrates epidermal necrosis (short thin arrow), congested dermal vessels (thick arrow), and calcification in small to medium-sized vessels in the deep dermis and subcutis (long thin arrow) (hematoxylin-eosin, original magnification x2)

**Figure 4:**
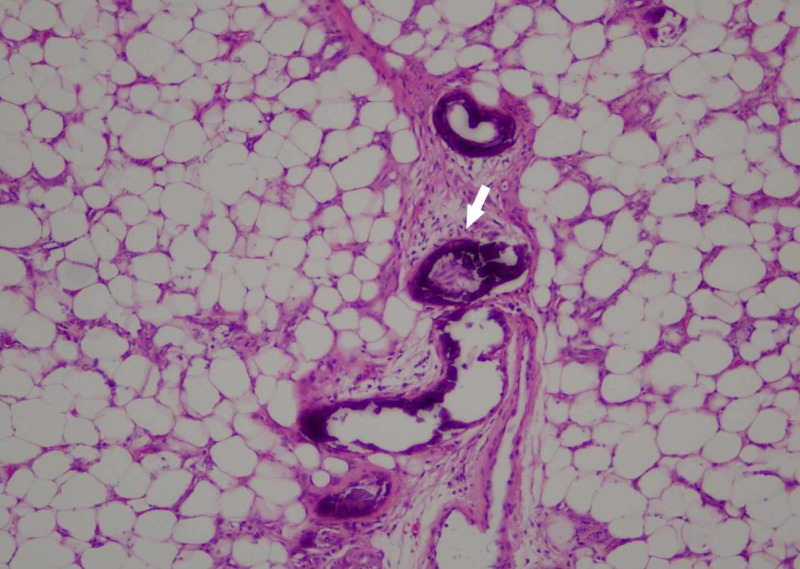
Vascular calcification (arrow) is noted in thickened septae between adipose lobules (hematoxylin-eosin, original magnification x20)

The clinical presentation of widespread skin necrosis and computed tomography (CT) findings of extensive calcinosis raised concern for calciphylaxis. The differential diagnosis included metastatic calcinosis, nephrogenic systemic fibrosis, and warfarin skin necrosis. A 6-millimeter punch biopsy of non-ulcerated lesional skin was performed and revealed gross calcifications. Histopathology demonstrated calcifications in small to medium-sized vessels in the deep dermis and subcutis, congested dermal vessels, and necrosis of the epidermis and adnexal epithelium, consistent with calciphylaxis (Figures 3-4).

Management included optimization of calcium and phosphorus levels as controlled by dialysis. Meticulous wound care and pain management with an interdisciplinary team were initiated. Treatment with intravenous sodium thiosulfate was deferred in the inpatient setting due to state government regulatory restrictions. As warfarin is a potential trigger for calciphylaxis and has been known to exacerbate it, the risks versus benefits of discontinuation of warfarin therapy were discussed with the patient. The WATCHMAN™ device (Boston Scientific, Marlborough, MA) was considered as an alternative to warfarin therapy; however, the patient ultimately opted for conservative management and discontinued the warfarin. The patient was transferred to hospice care per her wish and passed away a few months later.

## Discussion

Calciphylaxis is a rare, life-threatening cutaneous systemic disease. Patients with this condition present with painful livedo reticularis and violaceous plaques or indurated nodules which may blister; cutaneous necrosis often results in ulceration (Figure 1). Superimposed infection is a common complication.

Clinical history, physical examination, laboratory analysis, histology, and imaging are the main tools to exclude important differential diagnoses and confirm a diagnosis of calciphylaxis. Definitive diagnosis has traditionally necessitated a skin biopsy. The biopsy must include subcutaneous tissue for a proper diagnosis [2-3]. Diagnostic histopathology demonstrates medial calcification and intimal proliferation of small arteries, leading to ischemic epidermal necrosis and septal and lobular panniculitis (Figures 3-4) [4]. According to a retrospective case report, peri-eccrine calcium deposition, highly specific for calciphylaxis, was the only form of calcium deposition noted in four (7%) skin biopsies from patients with the disease [4]. Although subtle, peri-eccrine calcification may aid in the diagnosis of calciphylaxis in settings where typical vascular and extravascular calcification are not identified. The use of diagnostic stains for calcium phosphate deposits (e.g., von Kossa, Alizarin red) can highlight deposits not seen by routine light microscopy [5].

Many case reports, case-control studies, and observational studies have been published describing the predisposing factors for calciphylaxis which include: dependence on dialysis for more than two years, female gender, white race, obesity, diabetes mellitus, hypercalcemia, hyperphosphatemia, hypoalbuminemia, autoimmune conditions (such as systemic lupus erythematosus and rheumatoid arthritis), recurrent hypotension, and warfarin therapy. Hypercoagulable states, such as Protein C and S deficiency, antithrombin III deficiency, and antiphospholipid antibody syndrome, are also associated with this condition [1, 6-7].

A few case reports have been published on calciphylaxis associated with liver cirrhosis with or without the concurrent renal disease [8-10]. Liver cirrhosis, such as in the current case, can lead to decreased synthesis of coagulation factors and other proteins, including albumin, which can increase a patient’s susceptibility to injury. A low serum albumin level is a significant and strong risk factor for the development of calciphylaxis in the setting of chronic hemodialysis and cirrhosis [9].

Warfarin, a vitamin K antagonist, has been used for many years as an anticoagulant due to its action of inhibiting the carboxylation and activation of vitamin K-dependent clotting factors. Matrix Gla protein (MGP), a small Gla vitamin K-dependent protein, is the most powerful naturally occurring inhibitor of calcification in the human body. To become biologically active, MGP must undergo vitamin K-dependent carboxylation and phosphorylation. Vitamin K deficiency from dietary causes, or due to an acquired status as with warfarin therapy, can lead to the inactive desphospho-uncarboxylated form of MGP (dp-ucMGP). Subsequently, patients with vitamin K deficiency have an increased propensity for calcification. The association between circulating dp-ucMGP and cardiovascular disease, vascular calcification, renal function, and mortality in distinct populations have been reported [10]. 

Calciphylaxis is a lethal disease that carries high morbidity and an estimated six-month mortality of 50% [11]. Once the diagnosis is established, the formulation of a treatment plan with an interdisciplinary approach, including experts in multiple fields, should be considered [7]. Treatment involves wound management, analgesia, potential sodium thiosulfate therapy, management of mineral bone disease, adjustment of dialysis, and modification of risk factors [6-7]. Elimination of risk factors is an important component of the management of patients with calciphylaxis. Correcting hypercalcemia and hyperphosphatemia is recommended. The transition from peritoneal dialysis to hemodialysis is recommended; hemodialysis may advance wound healing when compared to peritoneal dialysis through better control of mineral metabolism [12]. Hypotensive episodes during dialysis sessions should be avoided, as hypoperfusion can delay wound healing and precipitate skin necrosis.

Calciphylaxis presenting with cardiac comorbidities, such as AF necessitating chronic anticoagulation, represents an intersection of medical conditions without clear treatment guidelines. Providing therapeutic anticoagulation in these patients is extremely difficult due to the simultaneous presence of underlying ESRD. In such cases, clinicians are often left balancing the morbidity and mortality risks inherent to calciphylaxis against those attributable to thrombotic and embolic events should anticoagulation cease [13]. Population-based studies suggest that AF occurs in approximately 20% of the patients with chronic kidney disease (CKD) who are not on dialysis and approximately 25% - 30% of the patients with CKD who are on dialysis [13-14]. As renal function declines, the prevalence of AF increases; among 235,818 subjects followed for six years, AF prevalence increased for those with an estimated glomerular filtration rate (eGFR) of < 30 ml/min/1.73 m^2^ compared with those with an eGFR of 30 to 59 ml/min/1.73 m^2^ [15]. A rigorous discussion of the risks and benefits of oral anticoagulation therapy (OAT), taking into account patients’ characteristics and preferences, is crucial to inform the decision. 

Although efficacy and safety outcome data is limited, to reduce the risk of stroke and systemic embolism for patients with non-valvular AF (except with moderate-to-severe mitral stenosis or a mechanical heart valve) with an elevated CHA2DS2-VASc score (**C**ongestive heart failure, **H**ypertension, **A**ge ( > 65 = 1 point, > 75 = 2 points), **D**iabetes, previous **S**troke/transient ischemic attack (2 points) vascular disease) and moderate-to-severe CKD, both the United States (US) Food and Drug Administration (FDA) and European Medicines Agency have approved reduced doses of both edoxaban and rivaroxaban in CKD patients with a creatinine clearance (CrCl) of 15 to 50 ml/min [16]. For apixaban, in patients with at least two of the following characteristics: age ≥ 80 years, body weight ≤ 60 kg, or serum creatinine ≥ 1.5 mg/dL, the recommended dose is 2.5 mg orally twice daily. The FDA has also approved the use of a specific low-dose dabigatran (75 mg twice daily) based solely on pharmacokinetic data for these patients [16]. For patients with AF who have a CHA2DS2-VASc score of 2 or greater in men or 3 or greater in women and who have end-stage CKD (CrCl < 15 mL/min) or are on dialysis, it might also be reasonable to prescribe dose-adjusted apixaban for oral anticoagulation [17].

**Figure 5:**
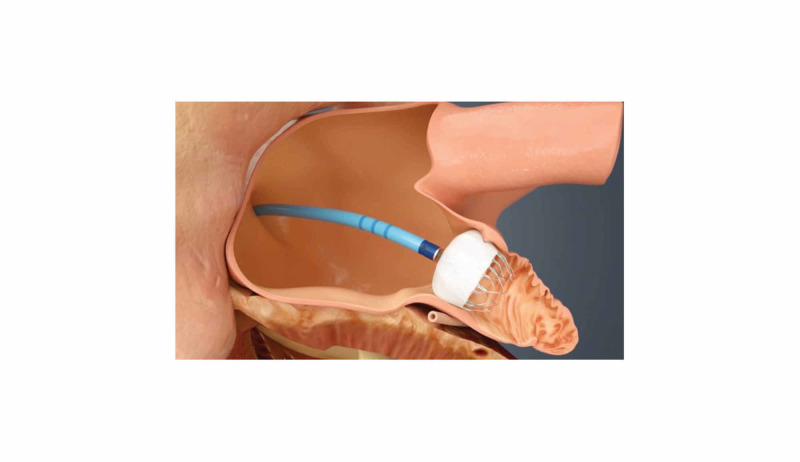
The WATCHMAN™ left atrial appendage (LAA) closure device is implanted in the left atrial appendage via a delivery catheter through a trans-atrial septal puncture *image used with permission from Boston Scientific

If it is deemed appropriate to use OAT in these patients, direct oral anticoagulants (DOACs) should generally be favored over warfarin therapy. Warfarin therapy is challenging in patients with renal disease as it can increase the risk of vascular calcification and cause acute kidney injury secondary to anticoagulant-related nephropathy (ARN) or warfarin-related nephropathy (WRN) [18]. In high-risk patients with non-valvular AF who are in need of an alternative to long-term OAT, an FDA-approved left atrial appendage (LAA) closure device, also called a WATCHMAN device, is a possible option for stroke risk reduction and should be considered (Figure 5) [17, 19-20].

## Conclusions

Calcific uremic arteriolopathy is a life-threatening disorder that must be considered in patients with renal insufficiency presenting with painful, non-healing skin lesions. Early recognition of patients at risk for developing calciphylaxis and a collaborative, multidisciplinary approach to eliminating or decreasing risk factors may avert the development of this disease. Specifically, for the significant number of patients with ESRD and/or cirrhosis requiring long-term anticoagulation therapy, such as those with AF, replacing warfarin with DOACs or novel interventions, such as an LAA closure device, may decrease pro-calcification and cardiovascular risks, thereby decreasing morbidity and improving the quality of life for patients at risk of calciphylaxis. Further studies are needed to identify the best treatment options for this rare disease.
